# Malignant melanoma mimic fungal infection a case report

**DOI:** 10.1186/s13000-022-01214-7

**Published:** 2022-02-27

**Authors:** Juan Sun, Yun-Zhu Zhu, Pan-Pan Shao, Jing Ke, Wei Wang, Qiu-Lin Sun, Jia-Bin Li, Jun Cheng

**Affiliations:** 1 Department of Science and Technology, Anhui University of Chinese Medicine, 230038 Anhui Province Hefei, P. R. China; 2 Department of Infectious Diseases, the First Affiliated Hospital of Anhui Medical University, Anhui Province 230022 Hefei, P. R. China; 3 Department of Infectious Diseases, Bozhou Peoples Hospital, 236803 Bozhou, Anhui Province P. R. China; 4 Pathology Department, the First Affiliated Hospital of Anhui Medical University, 230022 Anhui Province Hefei, P. R. China

**Keywords:** Melanoma, Pulmonary fungal infection, Biopsy, Misdiagnosis

## Abstract

**Background:**

Most of malignant melanomas originate from skin and often metastasize to the lungs, rarely metastasizes to the liver and bone. However, imageology characters of lung metastasis tumor are commonly similar to those of fungal infections.

**Case presentation:**

A patient was admitted with unhealed plantar puncture wound for 3 years, and cough and expectoration for 2 years. The chest computed tomography (CT) revealed multiple nodules with cavities, and the patient was diagnosed of pulmonary fungal infection in another hospital and received antifungal therapy for more than 8 months, but the clinical symptoms and chest imaging findings continue to progress. After admission, the pathological results of both lung biopsy and biopsy of the plantar wound 3 years ago indicated malignant melanoma.

**Conclusions:**

The diagnosis of lung lesions cannot rely solely on imaging diagnosis, lung biopsy should be performed if necessary.

**Supplementary Information:**

The online version contains supplementary material available at 10.1186/s13000-022-01214-7.

## Introduction

In recent years, the morbidity and mortality of melanomas showed increasing trend, and for most patients, metastasis had already occurred before diagnosis was confirmed. Lung is one of the most common site of melanoma metastases. Imageology, such as chest computed tomography (CT), is one of the most commonly used examination methods to confirm the diagnosis of lung diseases [1–4]. However, imageology characters of pulmonary melanomas are similar to those of pulmonary infection, especially fungal infection, both of which showing nodules or cavity-like changes [5–8]. Accordingly, it is often required to combine blood test results and pathological findings to make a clear diagnosis. Here, we presented a case of a middle-aged man with recurrent cough and expectoration, after receiving anti-fungal therapy for more than 8 months at a local hospital, finally diagnosed as melanoma with multiple metastases.

## Case presentation

On January 8th, 2021, a 58-year-old male patient was admitted to the Department of Infectious Diseases, the First Affiliated Hospital of Anhui Medical University due to recurrent cough and expectoration for 2 years and a significant deterioration in corresponding symptoms a month ago.

**Fig. 1:**
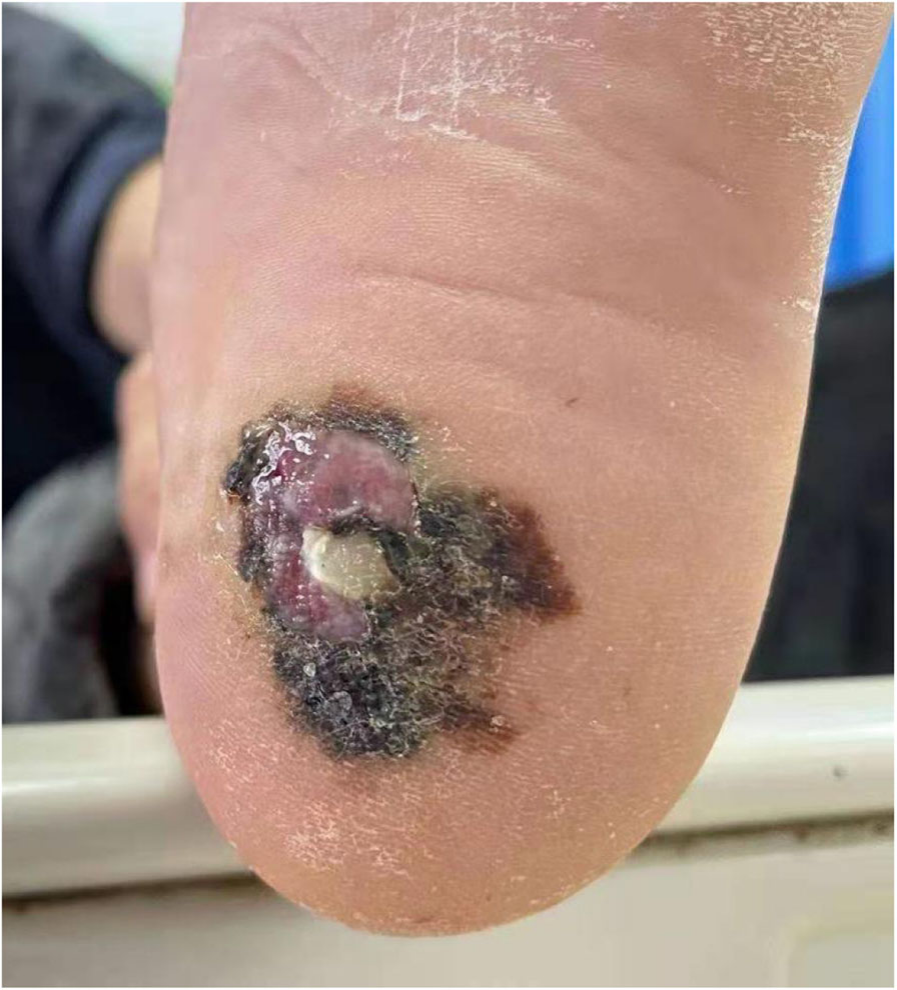
Plantar wound

**Fig. 2:**
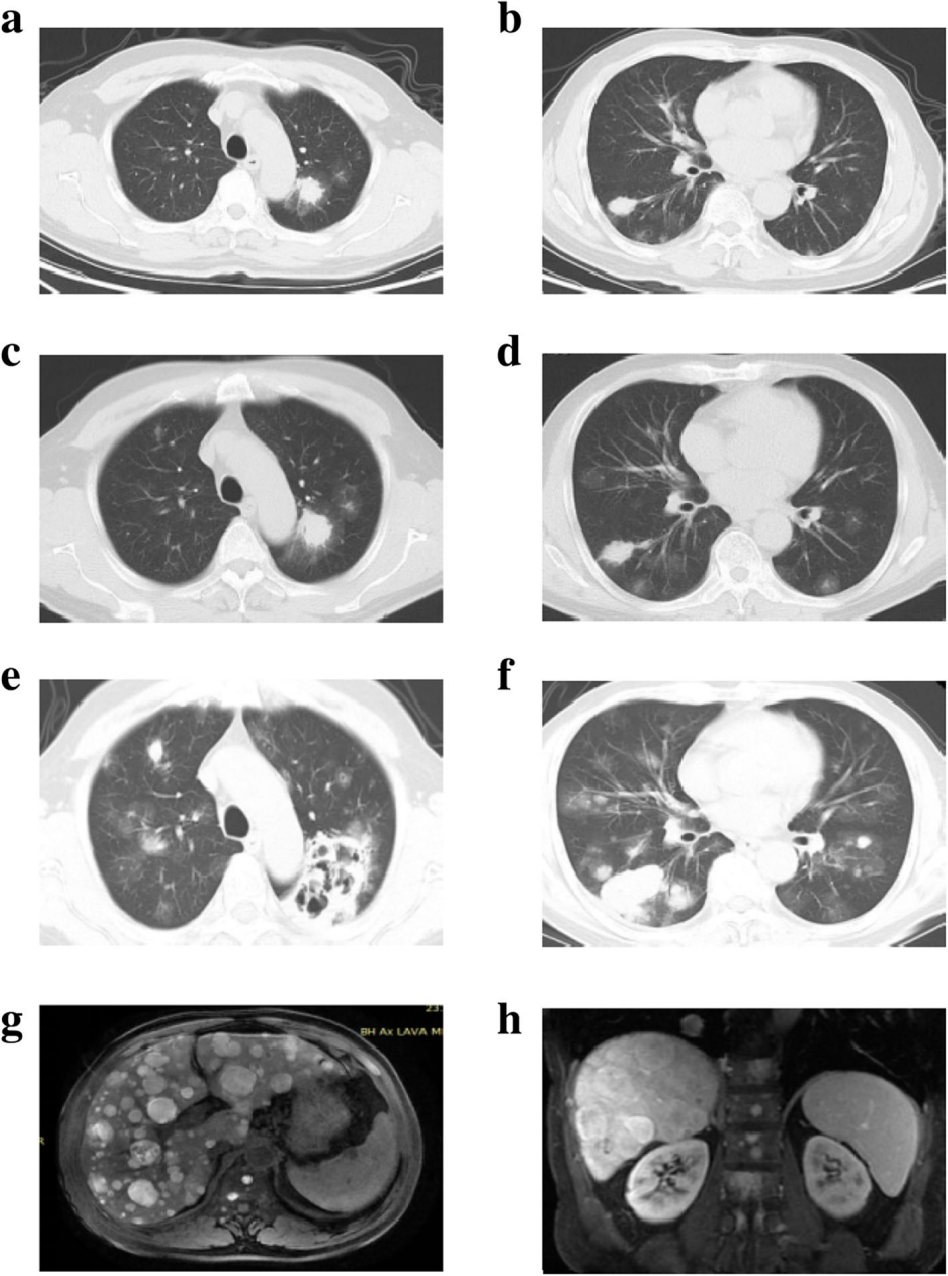
Relevant imageology characters during the patient’s visit. **A-B**, Chest CT on March 16th, 2020. **C-D**, Chest CT on June 2nd, 2020. **E-F**, Chest CT on Janurary 12th, 2021. **G-H**, Upper abdominal enhanced MRI on Janurary 10th, 2021

Medical history showed that on March 8th, 2020, at a local hospital, his chest CT revealed multiple abnormal density shadows in both lungs, indicating possible fungal infection. His left heel was punctured by an iron nail on a mushroom farm 3 years ago (Fig. 1). The wound remained unhealed with recurrent ulcer. Wound secretions from skin lesions were cultured and *Candida albicans* was identified. Gram-positive cocci and fungal spores were found using sputum smear microscopy. Fungal infection of the left heel accompanied by intrapulmonary dissemination was considered and fluconazole anti-fungal treatment was performed. Six months later, his symptoms of cough and expectoration were not significantly relieved, and chest CT result was worse than before (Fig. 2). And then, the anti-fungal agent was changed into voriconazole. The patient experienced pain in the right lower abdomen with obvious tenderness during coughing on December 25th, 2020. He went to the local hospital for further check. Multiple infectious lesions in the liver, liver abscess from some lesions, splenomegaly, and low density shadow of spleen were found using abdominal ultrasonography, indicating that there may be infections.

Accordingly, he was referred to our hospital. On admission, except for low fever, his vital signs were basically normal with body temperature at 37.8 °C, blood pressure at 109/84 mmHg, heart rate at 101 beat/min, and respiration rate at 23 time/min. No obvious abnormalities in lungs, soft abdomen, tenderness in the right lower abdomen, positive percussion pain in the liver area, and palpable spleen under the ribs were found through physical examination. An ulceration with an area of 4 cm × 5 cm was observed on his left heel. There were dark necrosis area surrounding the wound and bloody pus discharged from the center. Blood routine was basically normal with white blood cell counts of 7.10 × 10^9^/L, hemoglobin of 128.00 g/L, and platelet counts of 1.71 × 10^11^/L. Lower albumin level (31.80 g/L) and no significant abnormalities in liver and kidney functions were found. For inflammatory indicators, higher C-reactive protein (CRP) level (43.77 mg/L), higher erythrocyte sedimentation rate (ESR) level (56.00 mm/h), and no obvious increase in procalcitonin level (0.18 ng/mL) were observed. *Cryptococcus* capsular antigen test, G test, and GM test were all negative. Gram-positive cocci and gram-negative bacilli were found by sputum smear microscopy, while mycete and tubercle bacillus were not detected. Blood cultures were negative. For the tumor indicators, neuron-specific enolase (60.65 ng/mL) and ferritin (437.80 ng/mL) increased.

**Fig. 3:**
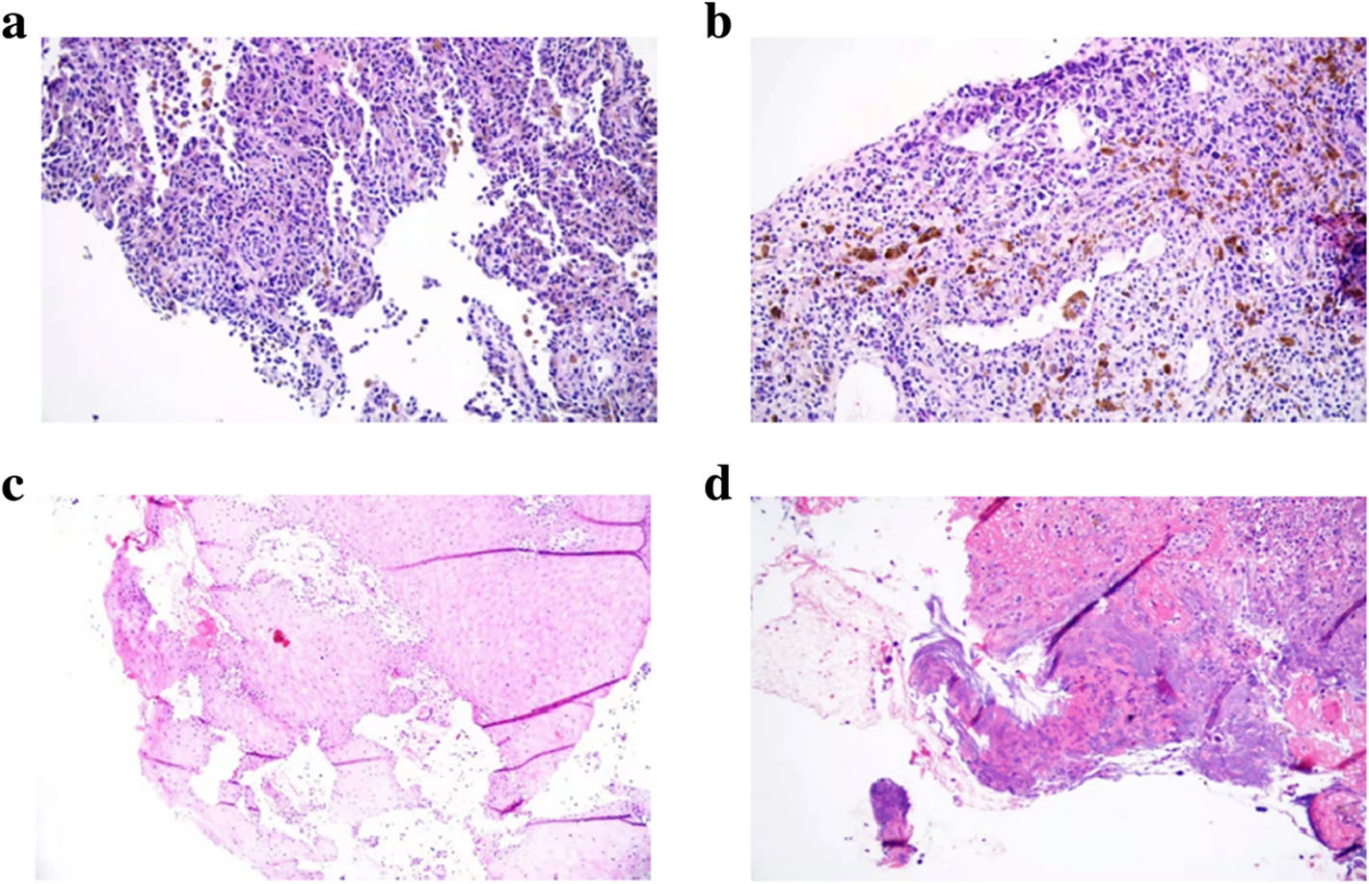
Pathological images. **A** and **B** were pathological images of lung biopsy. **C** and **D** were pathological images of plantar wound biopsy

He was preliminarily diagnosed as pulmonary fungal infection and suspected liver abscess after admission. Voriconazole was used for anti-infective treatment. Because he was unresponsive to anti-fungal therapy for 8 months, malignancy could not be ruled out. Lung and wound tissue biopsies were performed, and samples were sent for PACEseq metagenomic next-generation sequencing (mNGS) analysis (Hugobiotech, Beijing, China). Meanwhile, magnetic resonance imaging (MRI) showed multiple abnormal signals in both lungs, liver, thoracic and lumbar vertebrae, and multiple small and medium-sized lymph nodes in the retroperitoneum (Fig. 2). Chest CT plain scan plus enhanced scan indicated multiple nodules and clumpy high-density shadows in both lungs with blurred boundaries, and cloudy fuzzy shadows in the surrounding areas (Fig. 2). After enhancement, mild consolidation was observed and multiple cavities were visible inside. The above results of imageology indicated possible fungal infection. However, no pathogens were detected in lung biopsy tissues by mNGS. Accordingly, both pathological (tissue biopsy of both lungs and unhealed plantar puncture wound) and immunohistochemical results supported the diagnosis of malignant melanoma (Fig. 3). The liver and thoracolumbar lesions might be considered as results of melanoma metastases. However, he rejected our recommendation to perform liver biopsy. With consultation to the oncology department, it was concluded that his melanoma was accompanied by systemic metastases and immunotherapy might be feasible. However, considering the poor prognosis and the high cost, the patient and his family abandoned treatments and requested discharge after thorough considerations.

## Discussion

Melanomas originate from neural crest melanocytes, and are usually derived from benign growth-stunted mole. The pathogenesis of melanomas is associated with genetic and environmental factors, among which family history is the biggest risk factor. Other factors include ultraviolet radiation, skin photosensitivity, autoimmune state, and the presence of melanocytes or dysplastic moles [2, 4, 9, 10]. The pathogenesis of malignant melanomas is very complex, including a series of complicated interactions among external events and endogenous triggers, genetic mutations of BRAF, NRAS, and KIT, as well as endogenous and immune-related factors of the tumor [2, 10]. Approximately 90% of malignant melanomas originate from the skin, while melanomas of lung and liver origin are very rare [3, 11, 12]. In this report, tissue biopsy results of both lungs and unhealed plantar puncture wound indicate the patient should be diagnosed as malignant melanoma.

This patient had a plantar puncture wound 3 years ago and manifested pulmonary symptoms such as coughing and expectoration about 1 year later. However, without enough attention and proper care, concomitant infection lead to persistently unhealed wound. The wound was at the heel, which was repeatedly compressed and rubbed during daily activities. Under the combined actions of multiple factors, normal cells mutated into tumor cells, which gradually invaded into the dermis. Subsequently, unhealed wound can help the tumor cells get in touch with blood and lymphatic systems and gradually spread from the original site [9]. In this case, there were no mole cells in the plantar lesion, and the change of normal cells into tumor cells may be stimulated by the protracted wound, which was different from the published pathogenesis of melanomas [2].

Due to the highly invasive and metastatic characteristics of malignant melanoma, for most of patients, metastasis has already occurred before diagnosis is confirmed. The lung and pleura are the most common sites of melanoma metastases and usually the primary sites of initial metastasis, possibly due to the convergence of blood and lymphatic systems, and the peripheral blood pumped from the right heart through the pulmonary artery [4, 9]. Clinically, about 10-20% of the patients with malignant melanomas have concurrent liver metastasis [13], while 5-17% of the patients with stage IV melanomas have bone metastasis [14], which usually occur in the spine, pelvis, shoulder, and distal femur [15]. In this case, the patient has developed lung, liver, and thoracolumbar lesions, consistent with the published results.

For the patient in this case, it took nearly one year from the first visit due to cough and expectoration to the final diagnosis.The lung biopsy was not performed by the local doctor to confirm the diagnosis. Pathological diagnosis is the “gold standard” for pulmonary space-occupying lesions. For pulmonary fungal infection, scattered nodules, halo sign or crescent sign can be detected using CT scan [7, 8, 16, 17]. According to the diagnostic criteria specified by the European Fungal Research Organization for Cancer Therapy (EORTC), the crescent sign and the presence of cavities in pulmonary lesions are typical manifestations of pulmonary fungal infection [16].

In this case, the above characters were all detected using CT scan. Wound secretions from skin lesions were cultured by the local hospital and *C. albicans* was identified. Accordingly, it was considered as pulmonary fungal infection through skin lesions into the blood. However, lung biopsy was not performed at that time to confirm the diagnosis. For lung metastases, the most common manifestations are multiple pulmonary nodules, while pulmonary cavities may also occur [6, 7, 18]. Imageology characters of lung metastases are similar to those of pulmonary infection. Thus, it is quite difficult to make an accurate diagnosis only by imageology. The state-of-the-art mNGS technology would identify etiological pathogens, while the negative results might also provide useful information. In this case, the negative result of the lung biopsy further confirmed our diagnosis that the lung lesions were not caused by infection. In summary, before making a final diagnosis, the doctor should comprehensively evaluate clinical manifestations and examine the patient, especially the pathological results.

## Supplementary information

**Additional file 1**

## Data Availability

All data are fully available without restriction.
