# Supplementary figures and images for: Malignant melanoma mimic fungal infection a case report
Source: Diagn Pathol. 2022 Feb 27;17:33. doi: 10.1186/s13000-022-01214-7 (PMC8882303; doi:10.1186/s13000-022-01214-7)

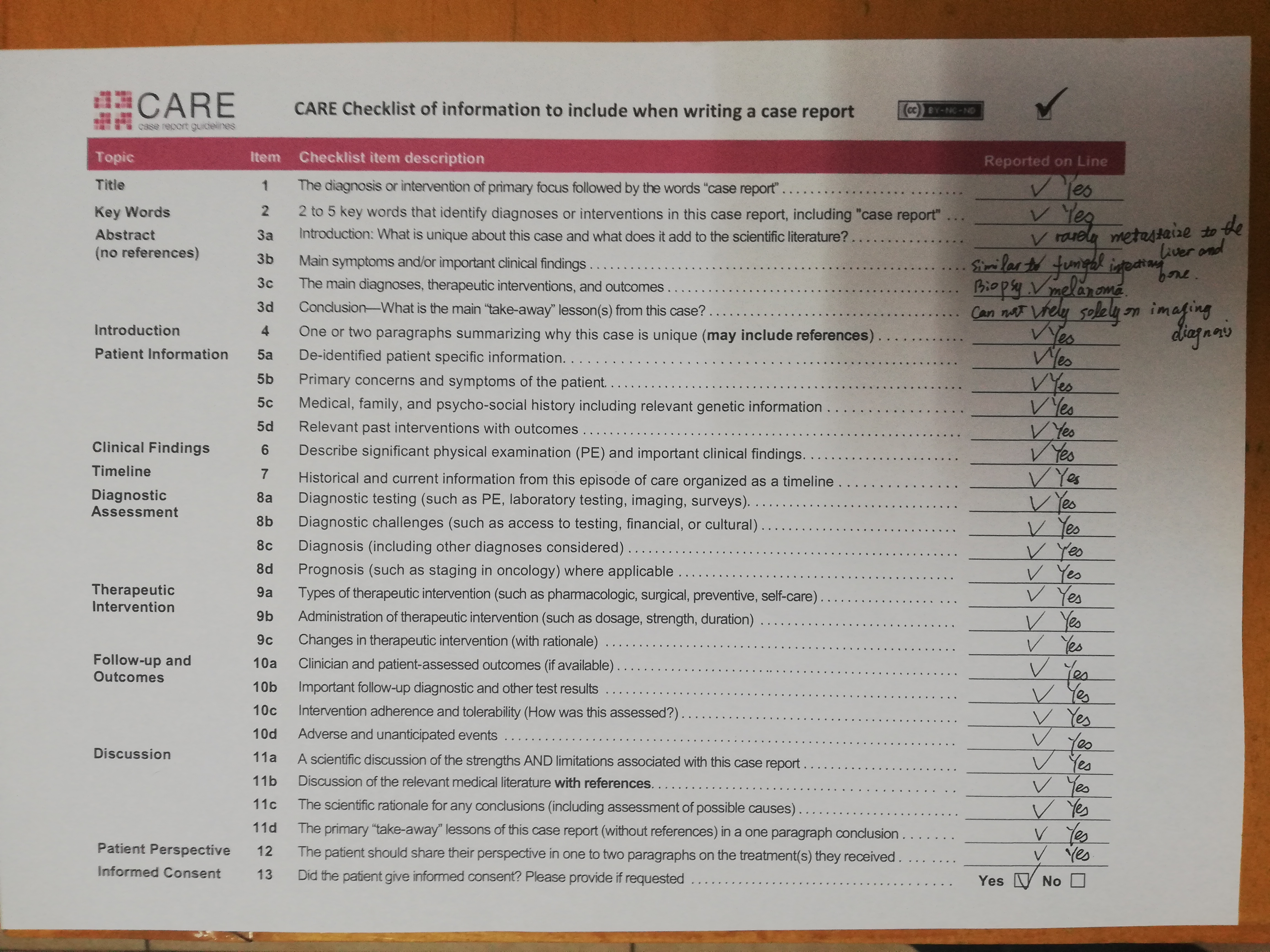

Supplement: Supplementary file 1 — Additional file 1 [file 13000_2022_1214_MOESM1_ESM.jpg]
